# Thermoelastohydrodynamic Characteristics of Low-Temperature Helium Gas T-Groove Face Seals

**DOI:** 10.3390/ma14112873

**Published:** 2021-05-27

**Authors:** Delei Zhu, Jing Yang, Shaoxian Bai

**Affiliations:** College of Mechanical Engineering, Zhejiang University of Technology, Hangzhou 310023, China; zhudlmail@163.com (D.Z.); bsx@zjut.edu.cn (S.B.)

**Keywords:** face seals, thermoelastohydrodynamic characteristics, low-temperature helium gas, T-groove

## Abstract

Thermoelastohydrodynamic lubrication behaviors of helium gas T-groove face seals are numerically simulated under conditions of low temperature and high pressure, with the consideration of real-gas properties including compressibility coefficient, viscosity, and heat capacity. It is found that helium gas T-groove face seal presents a sharp divergent deformation at low temperature and high pressure, which makes the opening performance weaken and the leakage rate increase. This result is obviously different from the case of high-temperature gas face seals. As the sealing temperature drops from 300 K to 150 K, the leakage rate increases about 17% and the opening force decreases about 15%. Moreover, with the growth of rotational speed, both the outlet film pressure and the sealing performance present a non-monotonic trend. Specifically, while the rotating speed of moving ring raises from 3000 to 30,000 r·min−1, the leakage rate changes more than 30%, and the opening force is reduced about 10%.

## 1. Introduction

Thermoelastohydrodynamic problems have always played an essential role in the precise design of gas face seals [1,2,3,4,5,6] and other mechanical elements [7,8,9,10] since both thermal distortion and elastic distortion significantly affect sealing performance. However, with the rapid development of pre-cool technology using low-temperature and high-pressure helium gas in hypersonic engines [11,12], there arouses a new thermoelastohydrodynamic lubrication problem, where not only the face distortions [13,14,15,16] but also the real-gas properties [17,18] become nonignorable, since compressibility coefficient, viscosity, and heat capacity vary obviously with decreasing temperature. Moreover, bidirectional groove structure, such as T-groove and elliptic-groove, has often been applied in gas face seals so as to effectively improve operational reliability under complex working conditions [19,20,21]. Therefore, the influence of the gas properties on thermoelastohydrodynamic characteristics of low-temperature helium gas T-groove face seals is significantly important.

In recent years, corresponding works about supercritical CO_2_ face seals have been conducted to investigate the real-gas property and it has a major impact on the thermoelastohydrodynamic performance of the seal. Fairuz’s analysis [22,23] shows that, when the operating condition approaches the critical point of CO_2_, the temperature, pressure, and density distributions of gas film change by 6.7%, 6.5%, and 39.5%, respectively, compared with the ideal gas. Du’s works [24,25] show that, compared with the air model in the supercritical zone, the opening force of the supercritical CO_2_ model enhances by about 3.3%, and the leakage rate decreases by about 20% when the sealing temperature is 940 K. Zhu’s research [26,27] demonstrates that the sealing property changes dramatically and the leakage rate increases about 4 times when the sealing temperature approaches the critical point of CO_2_. Moreover, Oike et al. [28,29] investigated the effect of the two-phase flow caused by the decreasing pressure on the sealing performance of the floating-ring seals at low temperature, and the experimental results showed that the leakage rate first decreases and then increases with the increase of the two-phase flow area. Zhang et al. [30,31] studied the sealing performance of the cryogenic face seal at different amounts of inlet pressure and rotational speed based on the experimental method and found that the leakage rate essentially does not change with the rotational speed at low temperature.

Although several studies about the sealing performance of the gas face seals have been carried out, the influence of gas property change on thermoelastohydrodynamic lubrication characteristics at low temperatures is still rare. Therefore, thermoelastohydrodynamic behaviors of helium gas T-groove face seals at low temperature and high pressure are analyzed in this study, considering the real-gas properties including compressibility coefficient, viscosity, and heat capacity. The face distortions, temperature distribution, pressure distribution, and sealing performance are numerally simulated under different basic film thickness, rotational speed, sealing temperatures, and sealing pressures. It provides theoretical support for engineering application and experimental research of gas face seals at low temperature.

## 2. Numerical Model

### 2.1. Geometrical Model

**Figure 1:**
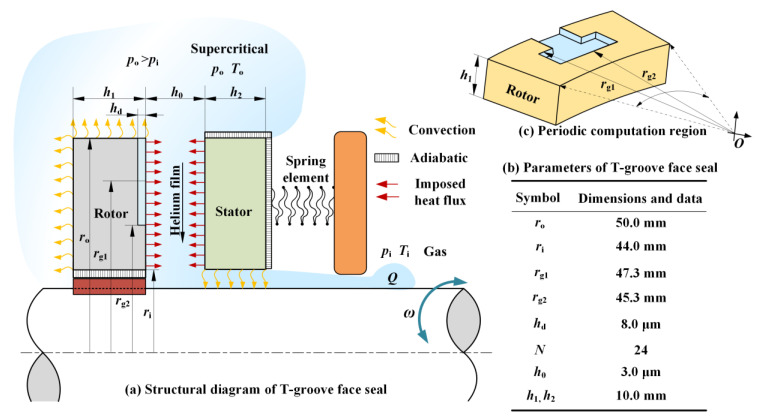
Geometry structure schematic diagram and thermal boundary conditions of the seals.

Figure 1 displays a typical structural diagram of the low-temperature T-groove gas face seal, where T-grooves with depth *h*_d_ are designed on the rotating ring. At rotational speed *ω*, the moving ring and the stationary ring of seal are separated from a clearance *h*_0_ by the opening force *F*, which induces a leakage rate *Q* along with the pressure gradient.

The thermal boundary condition between the gas film and the sealing surfaces is imposed heat flux, and the thermal boundary conditions between the sealing rings and the surrounding environment are convection and adiabatic. The T-groove is distributed periodically along the surface of the moving ring in the circumferential direction. As shown in Figure 1c, for the sake of simplifying the numerical solution process, a single T-groove is taken as the research object in the numerical calculation.

### 2.2. Governing Equations

For the analytical method of thermoelastohydrodynamic lubrication problem in steady state, the governing equations mainly include the real-gas state, Reynolds, solid heat conduction, and energy equation [32], which can be summarized as follows.

The Reynolds equation of the helium gas lubrication is shown below:

(1) $$\frac{\partial }{r\partial \theta }\left(\frac{h^{3}\rho }{\eta }\frac{\partial p}{r\partial \theta }\right)+\frac{\partial }{r\partial r}\left(\frac{h^{3}r\rho }{\eta }\frac{\partial p}{\partial r}\right)=6\omega \frac{\partial \left(\rho h\right)}{\partial \theta }+12\frac{\partial \left(\rho h\right)}{\partial t}$$

where *η* and *ρ* are the viscosity and the density of the helium, respectively.

**Figure 2:**
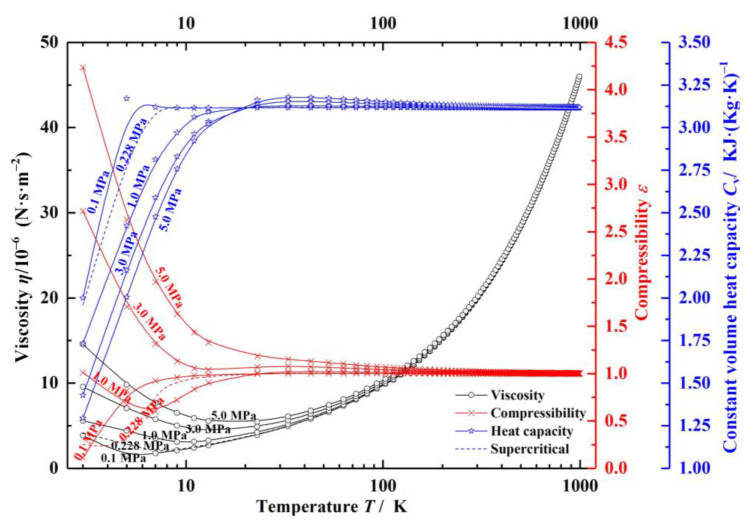
Variation of helium properties with different operating conditions.

The energy equation for helium gas lubrication can be expressed as

(2) $$\begin{array}{l} \left(-\frac{h^{3}}{12\eta }\frac{\partial p}{r\partial \theta }+\frac{\omega rh}{2}\right)\frac{\partial T}{r\partial \theta }-\frac{h^{3}}{12\eta }\frac{\partial p}{\partial r}\frac{\partial T}{\partial r}\\ =\frac{\eta \omega^{2}r^{2}}{h\rho c_{v}}-\frac{h^{3}}{12\eta \rho c_{v}}\left[{\left(\frac{\partial p}{r\partial \theta }\right)}^{2}+{\left(\frac{\partial p}{\partial r}\right)}^{2}\right]+\frac{k_{\mathrm{gs}1}}{\rho c_{v}}(T_{s1}-T)+\frac{k_{\mathrm{gs}2}}{\rho c_{v}}(T_{s2}-T) \end{array}$$

where *c*_v_ is the specific heat capacity, as shown in Figure 2.

The heat conduction equation for the static and rotating rings are respectively obtained as follows:

(3) $$\frac{\partial^{2}T_{s}}{r^{2}\partial \theta^{2}}+\frac{\partial }{r\partial r}\left(r\frac{\partial T_{s}}{\partial r}\right)+\frac{\partial^{2}T_{s}}{\partial z^{2}}=0$$

(4) $$\frac{k_{c2}}{\rho_{s2}c_{s2}}\left[\frac{\partial^{2}T_{s}}{r^{2}\partial \theta^{2}}+\frac{1}{r}\frac{\partial }{\partial r}\left(r\frac{\partial T_{s}}{\partial r}\right)+\frac{\partial^{2}T_{s}}{\partial z^{2}}\right]=\omega \frac{\partial T_{s}}{\partial t}$$

where *θ* is the direction of motion, *T*_s_ is the temperature of the static and rotating rings. 

The thermal boundary between the gas film and the solid is imposed heat flux, which satisfies the following equations:

(5) $$-k_{c1}{\left(\frac{\partial T_{s}}{\partial n}\right)}_{s}=k_{s1}\left(T_{s1}-T\right)$$

(6) $$-k_{c2}{\left(\frac{\partial T_{s}}{\partial n}\right)}_{s}=k_{s2}\left(T_{s2}-T\right)$$

where *k*_s_ is the convective heat transfer coefficient. 

For the above equations, considering the compressibility of real gas, the equation of state of pressure, *p*, can be described as

(7) $$p=\varepsilon c_{p}\rho i_{d}E_{m}$$

The gas film temperature equation is expressed as

(8) $$T=\frac{i_{d}E_{m}}{c_{v}}$$

where *ε* is the compressibility coefficient of the gas, *i*_d_ is the degrees of freedom of motion of gas molecules, *c_p_* is the pressure constant coefficient *(c_p_* = *R*/*c*_v_), *R* is the ideal gas constant, *E*_m_ is the energy of gas molecular per freedom. Here, the database REFPROP published by NIST is used to obtain the helium properties, and the properties are shown in Figure 2 [33]. 

As can be seen in Figure 2, with the pressure rising from 0.1 to 5.0 MPa, the compressibility coefficient of helium increases rapidly from 0.11 to 4.2, increasing by about 40 times at near-critical temperature, and the viscosity and specific heat capacity change by nearly 4 times and 1.5 times, respectively. With the increase of temperature, the influence of pressure on gas properties tends to be stable. The main factor affecting the gas properties is temperature. For the compressibility coefficient, when the temperature of helium rises by 10 K, the compressibility coefficient enhances by about 10 times at low temperature. 

The sealing performance parameters, mainly including leakage rate *Q* and opening force *F*, are respectively defined as:

(9) $$Q=\frac{h^{3}r\rho }{12\eta }\int_{0}^{2\pi }\frac{\partial p}{\partial r}d\theta$$

(10) $$F=\int_{0}^{2\pi }\int_{r_{i}}^{r_{o}}prdrd\theta$$

The moving ring of seal is made of stainless steel due to the high tensile strength at cryogenic temperature [34], and the stationary seal ring of Graphite due to the good self-lubricating property of Graphite material [35,36]. The material parameters of the seal ring are shown in Table 1. Through the coupling analysis of finite element method, the deformation of sealing surface is obtained, and the numerical mesh density of the sealing ring is 60 × 60 × 30. The gas film pressure distribution, temperature distribution, and seal ring temperature can be received by the finite difference method, and the numerical mesh density of the gas film is 60 × 60. The parameters of temperature field used in the numerical calculation of face seals are provided in Table 2. 

## 3. Numerical Results and Discussion

### 3.1. Temperature Fields

**Figure 3:**
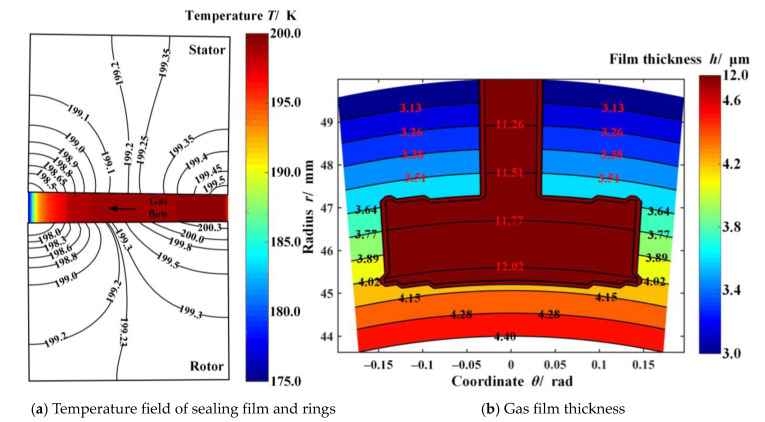
Cross–sectional temperature field and gas film thickness considering face distortions (*h*_0_ = 3.0 μm, *ω* = 20,000 r·min^−1^, *p*_o_ = 5.0 MPa, *T*_o_ = 200 K).

The cross-section temperature fields along the radial direction and the thickness distribution of gas film between seal rings are shown in Figure 3. Clearly, the film temperature gradually decreases along the direction of pressure flow, resulting in a temperature difference of 25.0 K between the outlet and inlet diameters (Figure 3a), since the gas expansion absorbs the heat. Meanwhile, the outlet temperature of the static and moving ring is 197.57 K and 192.75 K, respectively. The temperature difference between the outlet and inlet diameter of the seal ring is about 8.0 K and 2.0 K, leading to significant divergent face distortion (Figure 3b). The main reason for this phenomenon is the difference in convection heat transfer coefficient and thermal conductivity between the sealing rings. However, under the high-temperature condition, the face distortion of S-CO_2_ face seals is opposite, showing convergent deformation [27].

**Figure 4:**
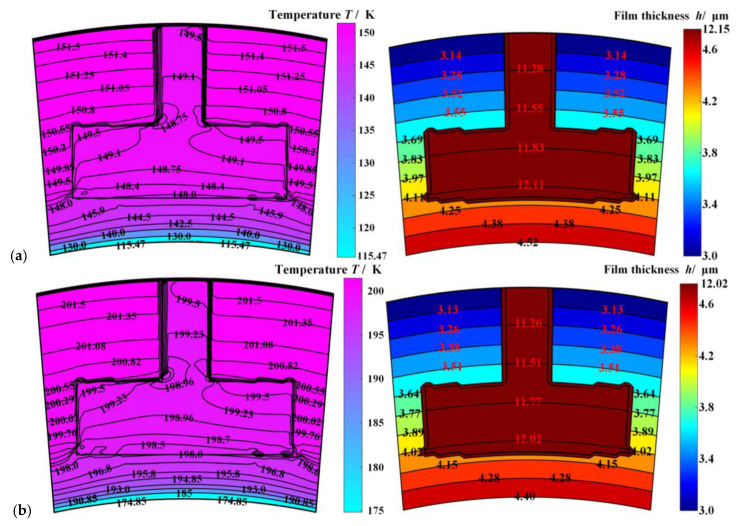

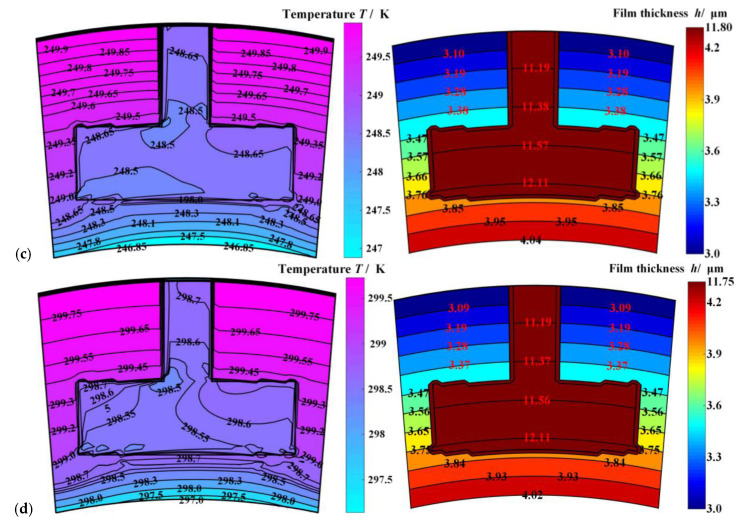
The film temperature fields and film thickness distribution of the sealing film under different inlet ambient temperatures (*h*_0_ = 3.0 μm, *ω* = 20,000 r·min^−1^, *p*_o_ = 5.0 MPa,). Note: (**a**) *T*_o_ = 150 K; (**b**) *T*_o_ = 200 K; (**c**) *T*_o_ = 250 K; (**d**) *T*_o_ = 300 K.

Further, temperature fields and film thickness distribution of the sealing film under different ambient temperatures are illustrated in Figure 4. Obviously, the gas film temperature drops about 3.0 K at the outer diameter, although the film temperature rises along the circular direction due to the velocity shear. With the rise of sealing ambient temperature from 150 K to 300 K, the gas film temperature difference drops from 30 K to 3 K. The reason is that with the rise of the sealing medium temperature, the compressibility of the sealing medium is reduced, then the heat absorbed by gas expansion decreases. More important is that, according to Figure 4, the film temperature drop leads to divergent face distortion. When the sealing ambient temperature is reduced by 150 K, the outlet gas film thickness increases by 0.5 μm. The reason is that the temperature difference between the outlet and inlet diameters is reduced, which makes the thermal deformation diminish. Simultaneously, it means there is a higher risk of dropping of the opening performance and contact wear failure [5,32].

### 3.2. Face Distortions

**Figure 5:**
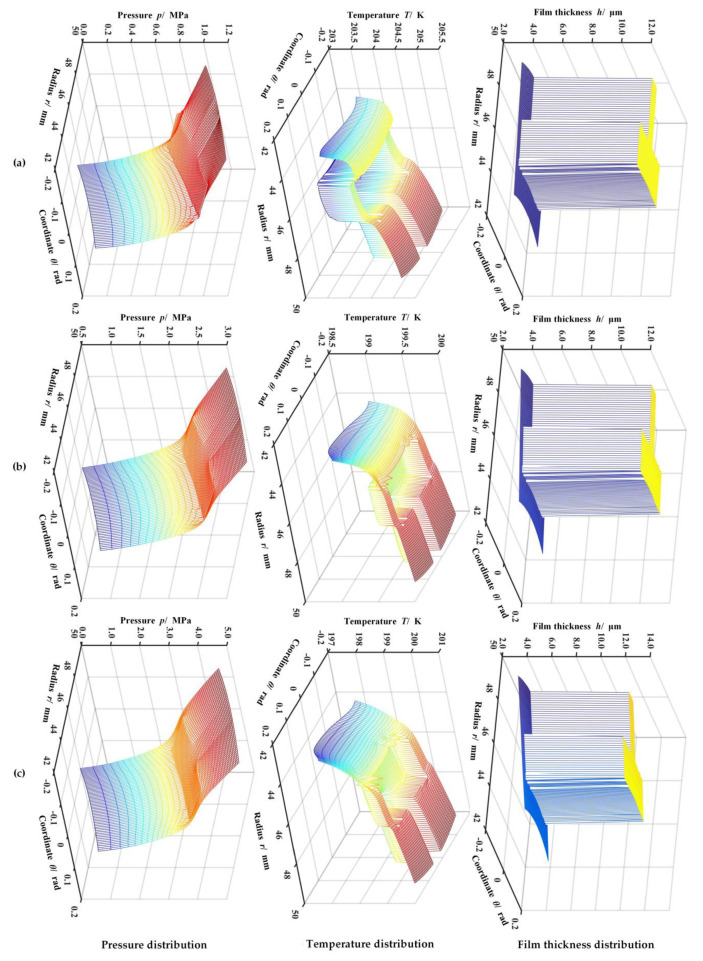
Variation of the pressure, temperature, and film thickness distribution of helium gas face seal with different inlet ambient pressure (*h*_0_ = 3.0 μm, *ω* = 20,000 r·min^−1^, *T*_o_ = 200 K). Note: (**a**) *p*_o_ = 1.0 MPa; (**b**) *p*_o_ = 3.0 MPa; (**c**) *p*_o_ = 5.0 MPa.

Figure 5 displays the gas pressure, temperature, and film thickness distribution at different sealing pressures. As seen in Figure 5a, with the sealing pressure rising, the hydrodynamic effect of T-groove decreases rapidly. Typically, the T-groove pumping effect drives the gas to flow, enhances the gas expansion rate, and then reduces the gas temperature. Meanwhile, the film temperature presents a decreasing profile in the radial direction as a whole because of gas expansion, although the velocity shear heat may cause the gas film temperature to rise. Thus, the interaction between the velocity shear heat and the pumping effect makes the calculation of the temperature distribution more complicated. While the sealing ambient pressure growth from 1.0 to 5.0 MPa, the outlet film temperature drops from 201.2 to 197.5 K (Figure 5b).

Different temperature gradients will produce different degrees of thermal deformation. Obviously, as shown in Figure 5c, the deformation of the sealing surface produces a divergence clearance at low temperatures and gradually enhances as the sealing pressure increases. When maintaining the ambient temperature as a constant of 200 K, the gas film thickness at the outlet will increase from 3.2 to 4.4 μm due to face distortion if the sealing pressure grows from 1.0 to 5.0 MPa.

### 3.3. Sealing Performance

**Figure 6:**
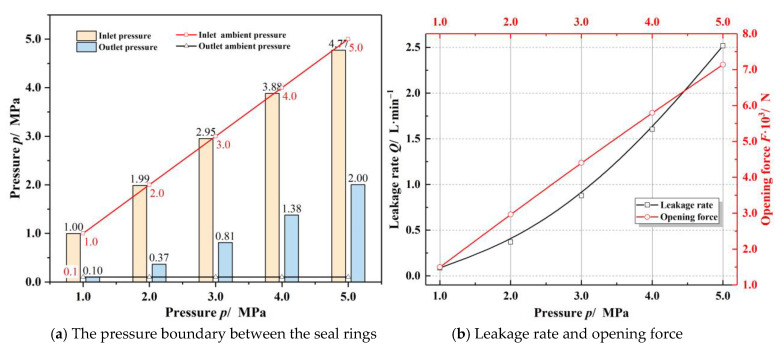
Influence of sealing pressure on the sealing performance (*h*_0_ = 3.0 μm, *ω* = 20,000 r·min^−1^, *T*_o_ = 200 K).

Figure 6 displays the influence of sealing pressure on sealing performance. As seen in Figure 6a, the inlet pressure loss and the choked flow effect are not apparent at low sealing pressure of 1.0 MPa, since the flow velocity of the gas at the outlet does not exceed the speed of sound. While the sealing ambient pressure is 5.0 MPa, the flow velocity of the gas at the outlet exceeds the speed of sound, the choked flow effect makes the outlet film pressure rise to about 2.0 MPa, much higher than the environmental pressure, while the inlet pressure loss is about 5%. The opening performance of the seal has a linear relationship with the inlet pressure. As a result, the opening force is enhanced about 5 times and the leakage rate increases nearly 29 times as the sealing ambient pressure rises from 1.0 to 5.0 MPa. 

**Figure 7:**
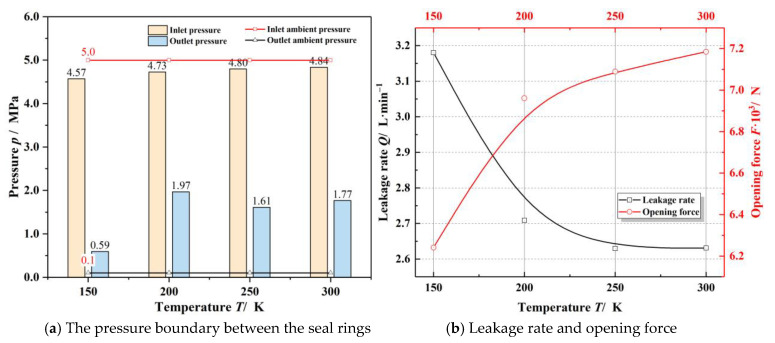
Variation of sealing performance under different sealing ambient temperature (*h*_0_ = 3.0 μm, *ω* = 20,000 r·min^−1^, *p*_o_ = 5.0 MPa).

Figure 7 presents the effect of the sealing ambient temperature on the sealing performance. As seen in Figure 7a, the inlet pressure loss increases while the outlet pressure presents a complex trend, with the decrease of the sealing temperature. The reason for this phenomenon is that it is easier for the gas flow speed to reach the sound velocity at low temperatures, resulting in more significant pressure loss. However, at low temperatures, since the sealing clearance presents a divergent shape, the gas flow velocity at the outlet becomes lower, and the blocking effect is weakened. Meanwhile, as the sealing temperature drops from 300 K to 150 K, the leakage rate increases about 17% as well as the opening force decreasing about 15%. The decrease of opening performance will reduce the gas film thickness and increase the probability of contact wear failure [2].

**Figure 8:**
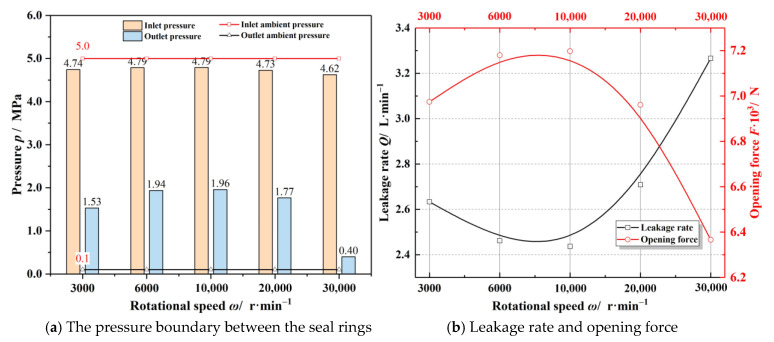
Influence of rotational speed on sealing performance (*h*_0_ = 3.0 μm, *p*_o_ = 5.0 MPa, *T*_o_ = 200 K).

The influence of the speed of moving ring on sealing property is given in Figure 8. With the rising of rotational speed, both the outlet film pressure and the sealing performance present a non-monotonic trend. In the cases of low speed, the outlet pressure increases with increasing speed. When the speed is large enough, the outlet pressure begins to decrease with the increasing speed. Correspondingly, when the speed is relatively low, the opening performance increases and the leakage rate decreases with increasing speed. Once the speed becomes large enough, the opening force reduces while the leakage rate increases. Here, while the rotational speed of the rotating ring increases from 3000 to 30,000 r·min^−1^, the leakage rate changes by more than 30%, and the opening force is reduced by about 10%. The numerical results of sealing performance are in accordance with the experimental results of Zhang et al. [30,31]. The experimental results show that the leakage rate fluctuates in a certain range with the increasing rotational speed, and the overall trend is increasing.

**Figure 9:**
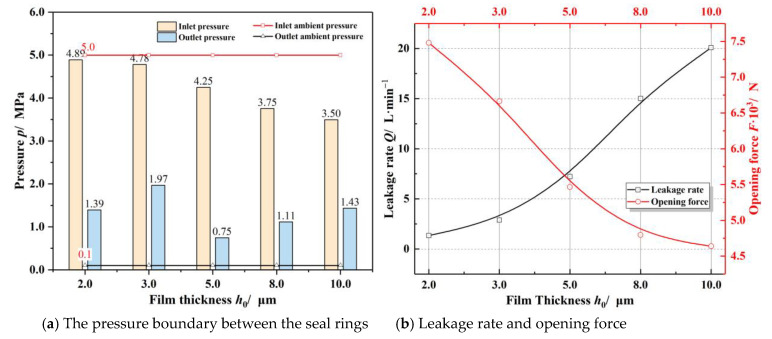
Influence of the basic film thickness on sealing performance (*ω* = 20,000 r·min^−1^, *T*_o_ = 200 K, *p*_o_ = 5.0 MPa).

Figure 9 illustrates the influence of the basic film thickness on sealing performance. As seen in Figure 9a, the high-speed airflow effect makes the gas film pressure boundary change significantly with rising basic film thickness, the inlet boundary pressure decreases rapidly, and the outlet pressure increases quickly. As a whole, the sealing performance presents a monotonic trend with the increasing film thickness at low temperatures. That is to say, as the basic film thickness increases, the pressure at the outlet and the leakage rate raise while the opening force and the inlet pressure decrease. For the face seals with groove depth of 8.0 μm, when the film thickness increases to 10.0 μm, the inlet pressure may drop from 5 MPa to 3.5 MPa, the outlet pressure may increase to 1.43 MPa, and the opening force decreases by about 38%.

## 4. Conclusions

The impact of real-gas properties on the thermoelastohydrodynamic lubrication of face seals become nonignorable. In this paper, the thermoelastohydrodynamic problem of helium gas T-groove face seals are investigated under conditions of low temperature and high pressure, with consideration of the real-gas properties. Based on the above numerical results, the following conclusions are drawn.

The opening performance of the seal has a linear relationship with the inlet pressure. The opening force enhances about 5 times and the leakage rate increases nearly 29 times as the sealing ambient pressure rising from 1.0 to 5.0 MPa.

Helium T-groove face seal presents a sharp divergent deformation at low temperature and high pressure, leading to an increase of leakage rate and a decrease of opening force. As the sealing temperature drops from 300 K to 150 K, the opening force decreases about 15% and the leakage rate increases about 17%. 

With increasing rotational speed, both the outlet film pressure and the sealing performance present a non-monotonic trend. Specifically, while the rotating speed of moving ring raises from 3000 to 30,000 r·min^−1^, the leakage rate changes more than 30%, and the opening force is reduced about 10%. The numerical results of the change trend of sealing performance are in accordance with the experimental results. Next, more attention should be focused on analyzing the influence of changes in gas properties on sealing performance at low temperature experimentally.

For the T-groove face seals with groove depth of 8.0 μm, when the film thickness increases to 10.0 μm, the inlet pressure may drop from 5 MPa to 3.5 MPa, the outlet pressure may increase to 1.43 MPa, and the opening force decreases by about 38%.

## Figures and Tables

**Table 1:** Parameters of ring materials.

Characteristics	Graphite	Stainless Steel
Materials density/kg·m^−3^	1800	7930
Young’s modulus/G·Pa	25	200
Poisson’s coefficient	0.2	0.3
Specific heat capacity/J·kg^−1^·K^−1^	710	500
Thermal conductivity/W·m^−1^·K^−1^	15	16.2
Linear thermal expansion coefficient/10^−6^ K	4	17.3

**Table 2:** Parameters of temperature fields.

Item	Symbol	Dimensions and Data
Convection heat transfer coefficient of the stationary and rotating ring at ambient boundaries/W·m^−2^·K^−1^	*k*_gs1_, *k*_gs2_	8.0
Thermal conductivity of gas/W·m^−1^·K^−1^	*k*_c-gas_	0.024
Degrees of freedom of motion of gas molecules	*i*_d_	3
Seal temperature/K	*T*_o_	150~300
Ambient pressure/MPa	*p*_0_	0.1
Ambient pressure/MPa	*p*_o_	1.0~5.0
Basic film thickness/μm	*h*_0_	2.0~10.0
Rotational speed/r·min^−1^	* ω *	3000~30,000

## Data Availability

The data presented in this study are available on request from the corresponding author.
